# Repeated Measures of Blood and Breath Ammonia in Response to Control, Moderate and High Protein Dose in Healthy Men

**DOI:** 10.1038/s41598-018-20503-0

**Published:** 2018-02-07

**Authors:** Lisa A. Spacek, Arthur Strzepka, Saurabh Saha, Jonathan Kotula, Jeffrey Gelb, Sarah Guilmain, Terence Risby, Steven F. Solga

**Affiliations:** 1 Department of Medicine, Johns Hopkins School of Medicine, 600 North Wolfe Street, Baltimore, MD 21205 USA; 2 Synlogic, 200 Sidney St #320, Cambridge, MA 02139 USA; 3 Department of Environmental Health Sciences, Johns Hopkins Bloomberg School of Public Health, 615 N Wolfe St, Baltimore, MD 21205 USA; 4 St. Luke’s University Hospital, Bethlehem, PA 18015 USA; 5 Present Address: Department of Medicine, University of Pennsylvania, 3400 Spruce Street, Philadelphia, PA 19104 USA; 6 Present Address: Thomas Jefferson University, Department of Medicine, 1025 Walnut Street, Philadelphia, PA 19107 USA

## Abstract

Ammonia physiology is important to numerous disease states including urea cycle disorders and hepatic encephalopathy. However, many unknowns persist regarding the ammonia response to common and potentially significant physiologic influences, such as food. Our aim was to evaluate the dynamic range of ammonia in response to an oral protein challenge in healthy participants. We measured blood and breath ammonia at baseline and every hour for 5.5 hours. Healthy men (N = 22, aged 18 to 24 years) consumed a 60 g protein shake (high dose); a subset of 10 consumed a 30 g protein shake (moderate dose) and 12 consumed an electrolyte drink containing 0 g protein (control). Change in blood ammonia over time varied by dose (p = 0.001). Difference in blood ammonia was significant for control *versus* high (p = 0.0004) and moderate *versus* high (p = 0.03). Change in breath ammonia over time varied by dose (p < 0.0001). Difference in breath ammonia was significant for control *versus* moderate (p = 0.03) and control *versus* high (p = 0.0003). Changes in blood and breath ammonia were detectable by fast, minimally-invasive (blood) or non-invasive (breath) point-of-care ammonia measurement methods. These pilot data may contribute to understanding normal ammonia metabolism. Novel measurement methods may aid research into genetic and metabolic ammonia disorders.

## Introduction

Ammonia physiology is important to numerous disease states including genetic metabolic diseases, *i.e*., urea cycle disorders and hepatic encephalopathy amongst patients with cirrhosis^1^. The biochemical pathways of ammonia genesis and disposal are understood. However, the relative contributions of various organs and body compartments including: the liver, the colon and its microbiome, the kidneys, muscle tissue, and small bowel enterocytes, to ammonia homeostasis is less well-defined. And, at the individual level, these knowledge gaps are magnified further. For example, basic information about the impact of a routine physiologic intervention, such as food consumption, is lacking.

These knowledge deficits impede clinical care. To illustrate using the common example of hepatic encephalopathy, patients with cirrhosis were, for many years, instructed to consume a low-protein diet on the assumption that this would reduce ammonia genesis and mitigate encephalopathy^2^. More recently, however, expert guidelines recommend the opposite to prevent sarcopenia^3^. These knowledge deficits impair the evaluation of new putative ammonia lowering therapies.

Clinical research in ammonia physiology has been difficult because ammonia is very reactive and is challenging to measure. Venous ammonia is most commonly measured but the standard protocol is subject to multiple random and nonrandom errors (*e.g*., tourniquet time, sweat contamination, blood cell hemolysis, and delay in laboratory measurement)^4^. Furthermore, the requirement for phlebotomy diminishes enthusiasm for studies requiring multiple samples. This is important because ammonia physiology, much like changes in blood glucose and many other metabolic processes, is dynamic.

Our aim was to evaluate the dynamics of the ammonia response to an oral protein challenge amongst healthy subjects. We hypothesized that ammonia would increase in a dose-dependent manner prior to returning to baseline. We quantified blood ammonia serially in response to three protein doses: 0 g, 30 g, and 60 g. We used the PocketChem BA blood ammonia checker (Lancashire, United Kingdom); this is a portable handheld device that uses a small volume (20 µL) blood sample^5^. In tandem, we also measured exhaled breath ammonia. Though the measurement of systemic ammonia through exhaled breath has great potential, breath measurement researchers must contend with a group of novel challenges including: variability in breathing, contamination with oral mucosal or bacterial ammonia, influence of ambient air, instrument humidity, and temperature^6^. Recently, we found that 60 g of oral protein significantly increased breath ammonia amongst 30 healthy subjects compared to a negative control day^7^. We demonstrated for the first time that mouth exhaled breath ammonia reflects systemic ammonia and examined novel and important biology. However, the study lacked blood ammonia measurement.

To our knowledge, the present study is the first to evaluate the dynamic ammonia response to control, moderate, and high oral protein doses and the first to do so using blood and breath ammonia measurement.

## Results

Twenty-two participants were studied and provided samples of breath and blood ammonia. Participants ranged in age from 18 to 24 years and were all men. Mean body mass index (BMI) was 24.8 kg/m^2^ (SD, 2.2; range, 21.6–31.6). Treatment groups included the control group (N = 12), moderate dose protein group (N = 10), and high dose protein group (N = 22). Mean blood ammonia ranged from 107 to 171 μg/dL at baseline and from 218 to 342 μg/dL at maximum levels (Table 1). And, mean breath ammonia ranged from 490 to 704 ppb at baseline and from 920 to 1642 ppb at maximum.

**Table 1:** Mean (+/−SD, standard deviation) and median (IQR, interquartile range) ammonia values for blood (μg/dL) and breath (ppb) at baseline and maximum by treatment group.

			Control (N = 12)	Moderate protein (N = 10)	High protein (N = 22)
Blood	Baseline	Mean +/− SD	143 +/− 58	107 +/− 30	171 +/− 69
		Median (IQR)	139 (95–194)	103 (85–126)	154 (112–212)
	Maximum	Mean +/− SD	218 +/− 82	232 +/− 84	342 +/− 87
		Median (IQR)	205 (157–245)	199 (176–280)	368 (267–400)
Breath	Baseline	Mean +/− SD	585 +/− 291	490 +/− 208	704 +/− 372
		Median (IQR)	682 (342–729)	514 (357–605)	625 (427–822)
	Maximum	Mean +/− SD	920 +/− 435	1107 +/− 543	1642 +/− 658
		Median (IQR)	1022 (556–1277)	982 (676–1442)	1550 (1250–2156)

**Figure 1:**
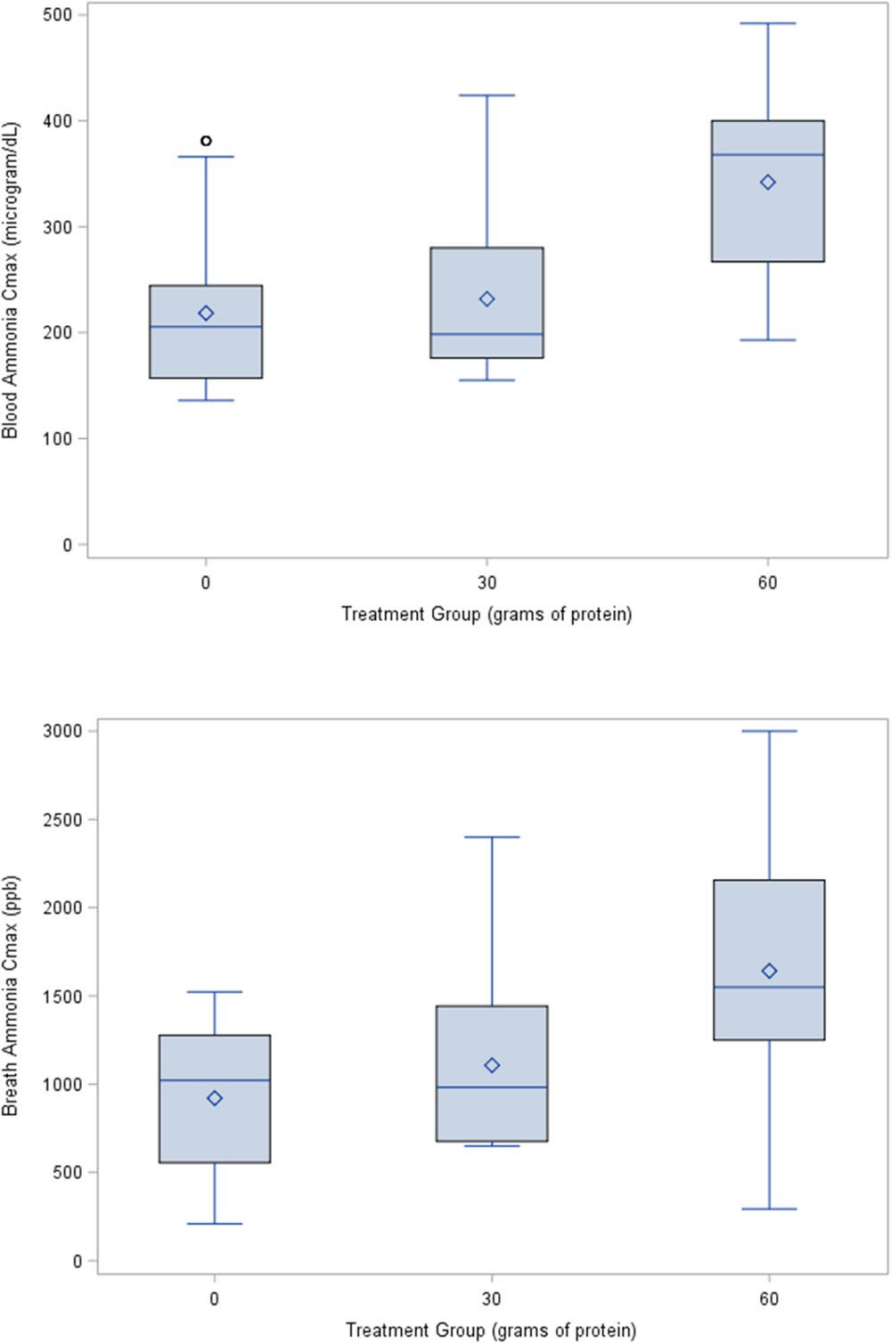
(**a**) Maximum blood ammonia (μg/dL) for treatment groups: control, 30 g protein, and 60 g protein. Box and whisker plot includes: mean (diamond), median (solid line), and range. (**b**) Maximum breath ammonia (ppb) for treatment groups: control, 30 g protein, and 60 g protein. Box and whisker plot includes: mean (diamond), median (solid line), and range.

**Figure 2:**
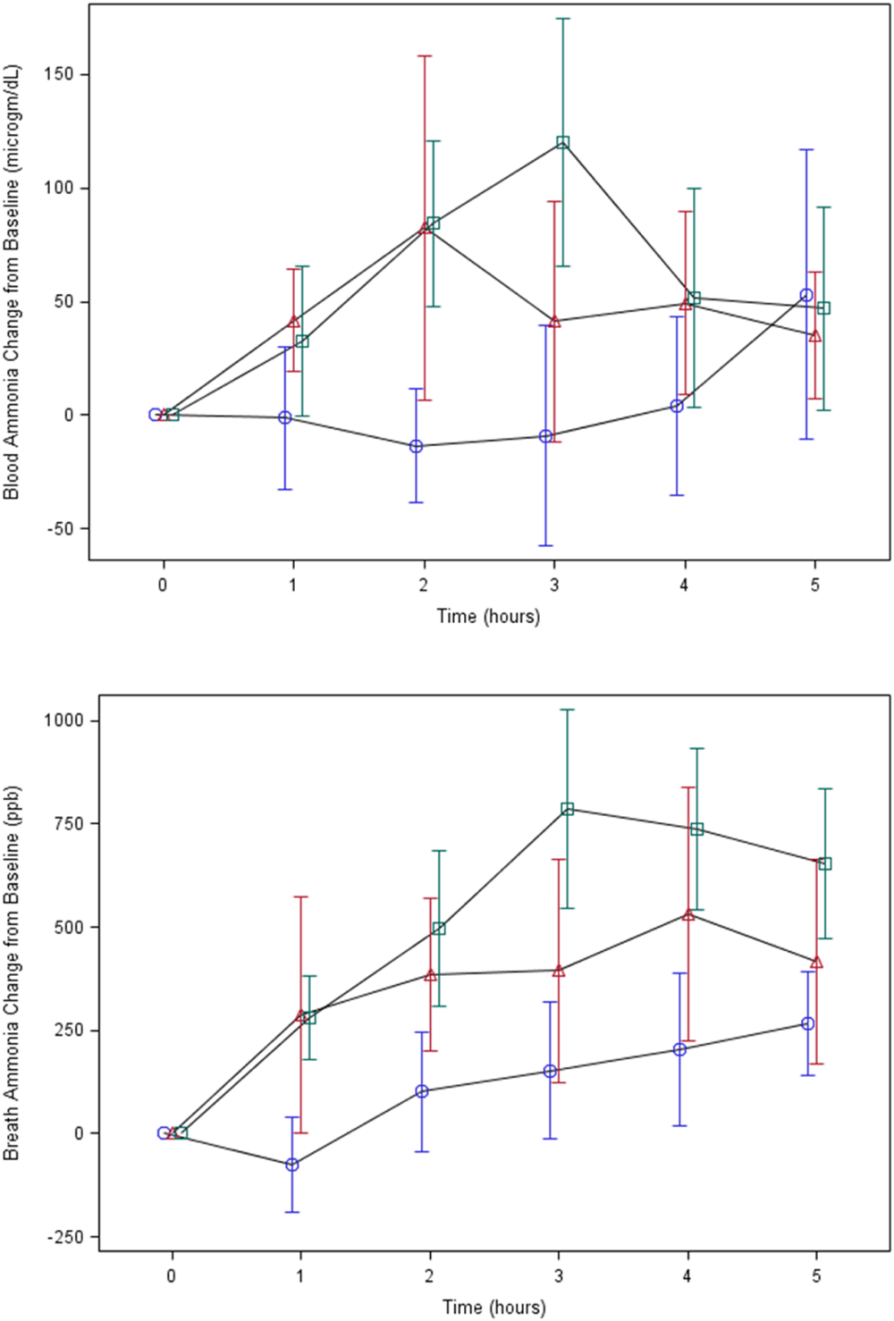
(**a**) Mean blood ammonia (μg/dL) change from baseline with 95% confidence interval by time (hours) for treatment groups: control (circle), 30 g protein (triangle), and 60 g protein (square). (**b**) Mean breath ammonia (ppb) change from baseline with 95% confidence interval by time (hour) for treatment groups: control (circle), 30 g protein (triangle), and 60 g protein (square).

Maximum concentrations (C_max_) of blood and breath ammonia by treatment groups (control, low dose, and high dose) are shown in Fig. 1a and b, respectively. Change from baseline over time in blood (Fig. 2a) and breath (Fig. 2b) ammonia varied by dose. Maximum levels for the high protein group occurred at 3 hours. Whereas maximum blood ammonia for the moderate protein group occurred at 2 hours, maximum breath ammonia for this group occurred at 4 hours.

Analysis of longitudinal data with a mixed model found that change in blood ammonia over time varied by dose (p = 0.001). Difference in blood ammonia was significant for control *versus* high (p = 0.0004) and moderate *versus* high (p = 0.03). As well, change in breath ammonia over time varied by dose (p < 0.0001). Difference in breath ammonia was significant for control *versus* moderate (p = 0.03) and control *versus* high (p = 0.0003).

## Discussion

We have demonstrated that ammonia increased after a 60 g oral protein challenge. Furthermore, this increase was detectable by fast, minimally-invasive (blood) or non-invasive (breath) point-of-care ammonia measurement methods. These pilot data can facilitate and inspire future study in protein metabolism for numerous purposes. These findings are novel and important. Only a single study published over 40 years ago has previously reported on the systemic ammonia response to an oral protein^8^. We believe that the research effort in ammonia physiology and protein metabolism has lagged at least in part because of the historical dependence on unreliable phlebotomy-based laboratory blood assays, and that our strategy offers new reason for optimism. Fast, minimal or non-invasive testing can help propel clinical research in cirrhosis, protein malnutrition and sarcopenia, hepatic encephalopathy, and pediatric urea cycle disorders.

Strengths of this study include different methods of measurement of ammonia over time and the comparison of ammonia levels in response to protein challenge. By measuring an individual’s response to a physiologic intervention, each individual serves as his or her own reference. Further, the measurement equipment is portable and offers real-time results. For example, we note that ammonia appears to increase toward the end of the testing period, even amongst the control group, which may be due to time of day influences previously described^9^. The connection between circadian clocks and metabolism may play a role. Recently, investigators found that short chain fatty acids directly modulate circadian clock gene expression within hepatocytes^10^.

Moreover, the relative tolerability of the measurements (*i.e*., lancet-derived blood samples and exhaled breath) enabled repeated measures within and across testing days. The tolerability of these tests, especially breath measurement, may enable clinical study in an adult or pediatric population. Finally, our conclusions are strengthened by the general agreement in results based on two measurement modalities from different body compartments (blood and breath).

Because of these assets, we can evaluate the hypothesis that oral protein increases systemic ammonia and outline its kinetics. This is important because even basic questions about food digestion and its relationship to ammonia metabolism remain unanswered. For example, a major recent review of ammonia metabolism^11^ cited five key papers on the impact of feeding on intestinal ammonia generation. Of these, 2 utilized animal models^12,13^, one utilized urinary protein measurement over multiple days and therefore could not assess the immediate impact of a food bolus^14^, and one was a small uncontrolled case series^15^. Only one article evaluated the response of blood ammonia and amino acid profiles to oral protein challenges in humans^16^. As demonstrated by our data, this study also found that, when an oral protein dose threshold was reached, systemic ammonia increased in healthy subjects and peaked 4 hours after ingestion.

Our study has limitations related to study design and study specifics. In that we evaluated the systemic ammonia response to an oral protein challenge, we did not investigate the sources of ammonia. Ammonia metabolism is complex and fluid, and its influences are not well understood at any level (cellular, organ, or person)^1^. The liver, for example, both removes and produces ammonia in different physiologic compartments (*i.e*., zone 1 *versus* zone 3 hepatocytes) under varying circumstances that remain poorly understood^17^. And, measuring specific organ flux is often highly invasive. For example, in a small study by Jalan *et al*., ammonia flux across the kidney was measured in multi-catheterized cirrhotic patients in response to a single challenge designed to simulate a gastrointestinal bleed^18^. Similarly, landmark studies evaluating the portal venous contribution to systemic ammonia have used multi-catheterized animals that were subsequently euthanized^19^ or humans at laparotomy without a protein challenge^20^. Hence, while our study design resulted in inherent limitations, we note these trade-offs are ubiquitous in ammonia research and indeed hope that our emphasis on fast, easy, point-of-care tests using a physiologic challenge helps overcome future study design challenges.

We speculate that the increases in systemic ammonia after an oral challenge found in this study may include increased gut bacterial ammonia production, increased amino acid degradation with subsequent increased systemic glutamine leading to increased renal ammonia genesis, increased renal ammonia genesis due to transient acidosis, some combination of these factors, or other mechanisms. Additionally, the source of breath ammonia increase may be distinct from the source of blood ammonia increase. For example, breath ammonia may have increased due to increased blood urea leading to increased salivary urea and oral ammonia genesis^21^. Lastly, we also note that our prior work demonstrated that an ammonia increase to an oral protein intervention is temporally associated with increases in breath hydrogen and acetone; the former serves as a unique gut transit timing marker, and the latter indicates amino acid degradation^7^. Hence, through simultaneous multi-metabolite measurement, breath analysis offers the potential to create a distinctive and more complete metabolic picture than standard blood assays.

Other important limitations relate to our study specifics. Though our control and moderate oral protein challenges were the same fluid volume, the control intervention had fewer calories. The control intervention was not a placebo and could have contributed to metabolic changes. Also, to reduce variables in this pilot, we used only a single protein source. It is unknown, therefore, if these results are generalizable. Further, we note that, because breath and blood measurements are both subject to significant variability and error, we did not find a significant correlation between them.

A final limitation is our use of only two ammonia measurement methods. There are multiple methods^22^ and testing platforms^23^ for measuring blood ammonia, and there have also been comparisons made between, for example, venous *versus* arterial blood ammonia, partial pressure NH_3_ *versus* NH_4_^+^^24,25^. Moreover, no recognized reference method, material, or timing interval exists. Further, researchers are still evaluating various subject factors and pre-analytical effects which may influence blood ammonia determination and interject error such as concomitant vitamin K ingestion and alanine aminotransferase (ALT) or gamma-glutamyl transferase (GGT) levels^26–28^. In general, however, the results of various ammonia measurement methodologies have been comparable under experimental conditions. But, as noted by Yurdaydin, “experimental conditions do not necessarily mimic the situation in patients”^29^. And, we believe that the research effort being applied to further refinements in laboratory-based blood testing has been slow, especially in relationship to the importance of unmet clinical need. Finally, lab-based methods requiring phlebotomy will always be limited due the inconvenience of sample acquisition.

The totality of this experience further underscores the need for novel, fast, accurate, point-of-care measurements easily tolerated by the test subject. We believe that the blood and breath methods used in this study are at least on par with the best blood and breath options available and have these key advantages. And importantly, there are many opportunities for future research. One approach would be to expand upon the present work by evaluating the impact, if any, of variables including individual factors (*e.g*., gender, age, race, body mass index, muscle mass), disease states (*e.g*., urea cycle disorders, cirrhosis), and oral challenges (*e.g*., protein and amino acid sources and compositions). Another approach would be to repeat a similar protocol and collect more metabolic data, such as standard urinary and blood markers for protein metabolism, to evaluate the sources of the ammonia increase and further validate the use of our ammonia measurement strategy.

## Methods

### Study Subjects

This study was approved by the Western Institutional Review Board. Participants were recruited *via* flyers and advertisements. All eligible participants provided written informed consent and all procedures followed the research protocols approved by the Western Institutional Review Board. Healthy volunteers, aged 18–40 years, without known periodontal, liver, or kidney disease or report of tobacco use, fasted 12 hours prior to presentation. Subjects with halitosis were excluded. Volunteers abstained from exercise the morning of the study and brushed their teeth at least 1 hour before arrival. Diabetics were excluded due to requirement that subjects present in the morning fasting. Subjects over 40 years old were excluded to minimize the risk of any subtle or undiagnosed metabolic condition.

### Study Protocol

Subjects presented after an overnight fast to a breath research office in Hellertown, PA. The breath research office is a dedicated, private research space. No other medical or business activities occur at this site. All subjects completed a survey to attest to: tobacco, antibiotic, valproic acid, investigational drug use in the past 30 days, known severe periodontal disease, liver or kidney disease, diabetes, recent change in diet or unusual (especially high protein) diet. For each subject, informed consent was obtained and baseline data including age, height, weight, and gender were documented. Baseline blood and breath ammonia were measured. Subjects then ingested a control (0 g), moderate protein (30 g) or high protein (60 g) oral challenge. Repeat measurements for blood and breath ammonia were measured hourly for 5 hours.

The total testing time was 5.5 hours (30 minutes to complete intake, consent, and baseline testing, then 5 hours of post oral challenge testing). We chose 5 hours based on prior experience with high protein challenges and intended to capture maximum ammonia levels. No water, mouth rinse, tooth brushing, or chewing gum was permitted in the hour prior to the first breath collection. Water ingestion was allowed for 10 minutes after any one of the hourly breath collections.

Safety and tolerability was assessed throughout, and subjects were called the day following testing to ask about any adverse events.

The oral challenge for moderate or high protein testing days consisted of a commercially available shake (Premier Protein). Each shake (325 mL) contained 160 calories: 30 g protein, 3 g fat, and 5 g carbohydrate. For the moderate and high protein testing days, a subject consumed one or two shakes, respectively, for a total of 30 g or 60 g protein. Per the package label, this is a complete protein comprised of milk protein concentrate, calcium caseinate, and whey protein concentrate. For the control day, subjects consumed a commercially available electrolyte drink (650 mL of Gatorade) containing zero protein; this was the same control used in an earlier study and was provided to allow for non-protein calorie intake in fasted subjects. All subjects completed the oral ingestion within 15 minutes.

All 22 subjects first completed the high protein testing day (60 g). This was to determine whether an ammonia response signal was detectable. Once confidence was established in this conclusion, then subjects were invited to complete the control (N = 12) and moderate (N = 10) protein test days.

### Determination of Blood Ammonia

Blood ammonia was determined using the PocketChem BA blood ammonia analyzer obtained from Woodley Equipment (Lancashire, United Kingdom). PocketChem BA uses a microdiffusion method based on alkaline liberation of gaseous ammonia followed by passage through a spacer, and reaction with a bromocresol green indicator strip followed by colorimetric quantification of ammonia level. This analyzer uses a single wavelength (635 nm) reflection measurement method^30^. This small portable device uses 20 *µ*L of blood collected with a disposable capillary tube and pipette. Alcohol wipes were used to cleanse the site thoroughly and a lancet was used to obtain the blood sample. Its methodology has been previously described and it favorably compared to other blood ammonia measurement methods^5,31,32^. The precision, linearity, repeatability, and accuracy of the device have been evaluated^33^. It is used routinely in patient care and clinical research^34,35^ around the world, though it is not licensed for patient use in the United States.

### Determination of Breath Ammonia

Breath was determined using a prototype portable ammonia monitor provided by Bedfont Scientific, Ltd (Kent, UK). The basis of the ammonia measurement is thermal oxidation of breath ammonia to nitric oxide (with a minimal amount of nitrogen dioxide) and water. This quantitative conversion occurs in real-time. The thermal catalytic oxidation of breath ammonia to nitric oxide was first reported by Larson *et al*. in 1979^36^. These investigators used a modified commercial environmental chemiluminescent nitric oxide analyzer (Model 14D, Thermo Electron Corp., Waltham, MA) to perform their studies. This approach to measure ammonia via thermal oxidation to nitric oxide has been adopted commercially and there are currently at least four instruments (Model 17 C, Thermo Electron Corporation, Franklin, MA; Model 17*i* Thermo Fisher Scientific, Waltham, MA; Model ML9842, Casella Monitor, Bedford, UK; and Model T201E, Enviro Technology Services, Gloucestershire, UK) that quantify environmental levels of ammonia using chemiluminescent detection of nitric oxide. The determination of environmental concentrations of ammonia using these commercial instruments have been validated by the U.S. Environmental Protection Agency (EPA) in the Environmental Technology Verification (ETV) Program.

In the studies reported herein, the oxidation of ammonia is thermally cracked at 750 °C and a portion of oxidized breath is extracted at a constant rate (150 mL/min) and passed through three electrochemical sensors that analyze nitric oxide, oxygen and hydrogen. Monitoring nitric oxide with an electrochemical sensor instead of chemiluminescent monitor increased the portability of the resulting instrument. Additionally, the use of an electrochemical sensor for oxygen allows the end-tidal oxygen concentration to be monitored and the resulting breath ammonia data can be normalized to physiology. The plateaus for the exhalation of breath ammonia correspond to stable plateau for lowest concentration of oxygen. This breath ammonia monitor adopts the accepted American Thoracic Society/European Respiratory Society (ATS/ERS) real-time protocol to measure the concentration of nitric oxide (F_e_NO) that originates from the respiratory tract. The study subject is seated and asked to exhale into a tube containing a flow restrictor while maintaining a constant mouth pressure of 10 cm of H_2_O^37^. This protocol was followed to reduce any variability of endogenous breath nitric oxide since the selected mouth pressure ensures that the velum is blocked, thereby minimizing any contributions from the nose and sinuses to the levels of nitric oxide originating from the airways. The flow restrictor and selected mouth pressure produces the required exhalation flow rate of 50 mL/s. Controlled exhalation rate is used since the production of breath nitric oxide in the airways is flow dependent. Mouth pressure is displayed visually and is used to prompt the individual to breathe at the defined constant rate of exhalation. The individual is encouraged to exhale at the constant exhalation rate to achieve a plateau in the nitric oxide (ammonia) *versus* time profile of at least 3 seconds. This single breath maneuver is repeated until the measured nitric oxide (ammonia) plateaus fall within 10% of the mean value. Additionally, adopting the ATS/ERS protocol reduces the variability of the breath ammonia in longitudinal breath samples so intervention studies can be conducted.

**Figure 3:**
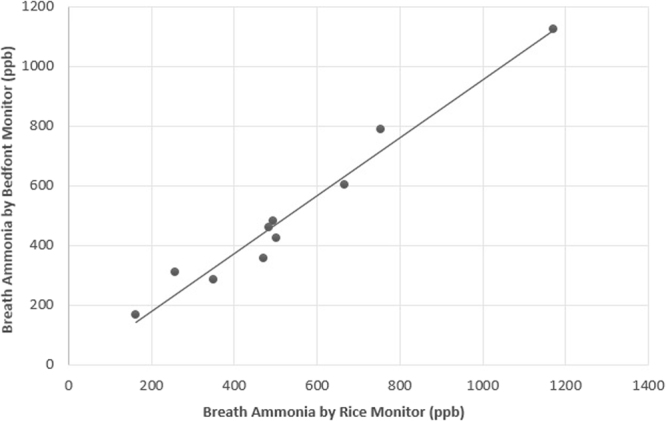
Correlation analysis of ammonia measurement (ppb) by Bedfont monitor versus Rice monitor. Solid line represents regression line. Bedfont = −15 + 0.97 × Rice; r^2^ = 0.97; Pearson correlation coefficient = 0.98. N = 10; Graphed are means of three samples for each.

The electrochemical sensors used in this prototype instrument are found in many commercial breath monitors to measure nitric oxide, oxygen, and hydrogen. The addition of the hydrogen sensor allows the studies of the origin of ammonia to be performed. The monitor was calibrated daily and provides a reading in parts per billion (v/v). The accuracy of this prototype monitor was compared to an existing monitor developed by colleagues at Rice University based on the absorption of mid IR radiation^6,7,38,39^. Figure 3 illustrates a scatterplot of means of 3 duplicate samples obtained from 10 individuals by the Bedfont and Rice breath ammonia monitors. As described by the ATS/ERS recommendations for standardized procedures for the measurement of exhaled lower respiratory nitric oxide^37^, exhaled ammonia was calculated as the mean of three values. Of the 60 total samples, 2 were excluded based on ATS/ERS protocol as a third measurement that did not agree within 10% of the other 2 measurements. Correlation analysis of ammonia measurement (ppb) by Bedfont monitor *versus* Rice monitor is represented by regression line, Bedfont = −15 + 0.97 × Rice, with r^2^ of 0.97 and correlation coefficient of 0.98.

Breath and blood measurements were obtained within a period of 2 minutes.

### Statistical Analysis

For each participant, blood and breath ammonia concentrations collected at 6 time-points (at baseline then every hour for 5 hours) were used to calculate change from baseline, fold change, maximum concentration achieved, and area under the curve. For each of the three treatment groups: control (0 g), moderate protein (30 g), and high protein (60 g), the following parameters were calculated at each time point: mean ammonia concentration, mean change from baseline, and mean fold change. Differences in mean blood and breath ammonia amongst the three treatment groups were compared.

Longitudinal analysis was performed by linear mixed-effects model analysis (PROC MIXED, SAS, Cary, NC) and included fixed effects of baseline ammonia, treatment group (protein dose), time, and interaction of protein dose and time^40^. The inclusion of an interaction term allowed for the evaluation of the relationship between treatment dose and elapsed time on the measured amount of ammonia. Time was treated as a categorical variable. Repeated measures and baseline values for each participant were treated as random effects. We evaluated multiple covariance structures for best fit based on relative goodness of fit of the covariance structures and designated compound symmetry as the covariance type. Pairwise comparisons were performed using estimate statements with contrasts from the longitudinal model.

All collected data were analyzed and no data were missing. All statistical analyses were conducted with SAS version 9.3 (SAS Institute, Inc., Cary, NC). Hypothesis tests were two-tailed and used a 5% significance level.

### Data Availability

The datasets generated during and/or analyzed during the current study are available from the corresponding author on reasonable request.
